# The fading boundaries between patient and environmental routes of triazole resistance selection in *Aspergillus fumigatus*

**DOI:** 10.1371/journal.ppat.1007858

**Published:** 2019-08-22

**Authors:** Jochem B. Buil, Rasmus K. Hare, Bas J. Zwaan, Maiken C. Arendrup, Willem J. G. Melchers, Paul E. Verweij

**Affiliations:** 1 Department of Medical Microbiology, Radboud University Medical Center, Nijmegen, the Netherlands; 2 Center of Expertise in Mycology Radboudumc/CWZ, Radboud University Medical Center, Nijmegen, the Netherlands; 3 Unit of Mycology, Statens Serum Institut, Copenhagen, Denmark; 4 Laboratory of Genetics, Wageningen University and Research, Wageningen, the Netherlands; 5 Department of Clinical Microbiology, Rigshospitalet, Copenhagen, Denmark; 6 Department of Clinical Medicine, University of Copenhagen, Copenhagen, Denmark; Geisel School of Medicine at Dartmouth, UNITED STATES

*Aspergillus fumigatus* is a saprobic fungus that may cause allergic syndromes, chronic pulmonary aspergillosis (CPA), and acute invasive aspergillosis (IA). Many patients suffering from aspergillus diseases benefit from antifungal therapy. Itraconazole, voriconazole, posaconazole, and isavuconazole have been shown to be the most effective compounds for prevention and treatment of the various aspergillus diseases. The use of alternative antifungal drugs, i.e., liposomal amphotericin B, is limited by toxicity and the echinocandins by fungistatic activity, while both also require intravenous access. As a consequence, the triazoles have become the recommended option for first-line therapy and chemoprophylaxis [1,2].

Unfortunately, the effective use of triazoles has been threatened by the emergence of resistance in *A*. *fumigatus* [3]. In voriconazole-treated patients, day 42 survival was 21% lower in voriconazole-resistant IA compared with voriconazole-susceptible infection [4]. As the number of available drug classes is already very limited, some aspergillus diseases, such as central nervous system IA, are virtually untreatable if caused by a triazole-resistant isolate.

## How is triazole resistance selected in *A*. *fumigatus*?

Triazoles represent a stress factor to the fungus that can be overcome by genetic adaptation, i.e., the acquisition of heritable modifications through natural selection, enabling the fungus to survive and reproduce in the azole-containing environment. Mutation is thus an important requirement for adaptation as genetic variations are created that might be better suited to survive in the presence of azoles. *A*. *fumigatus* can benefit from three reproduction modes that create this variation [5]. Asexual sporulation allows spontaneous mutations to occur in each one of the many produced spores, while, in addition, extensive genetic reshuffling takes place through recombination during sexual and parasexual reproduction. Asexual sporulation is abundant in *A*. *fumigatus*, but the frequency and conditions that support (para)sexual reproduction remain largely unknown [5]. When *A*. *fumigatus* is exposed to triazoles in experimental conditions, resistance mutations may emerge both through asexual reproduction and sexual reproduction [6], indicating that both modes enable adaptation to the azole environment. Resistance generally comes with a fitness cost and reduced growth rates are observed in resistant isolates recovered from triazole-treated patients when grown in the absence of triazoles [5,7]. Although these isolates have an advantage in the azole environment, they will generally not be able to compete with wild-type isolates in the absence of triazoles. Experimental evolution experiments, however, showed that resistant isolates often undergo compensatory evolution when growing in the azole-free environment, thus compensating for the fitness cost [5]. These evolutionary trajectories ultimately benefit the fungus as it enables *A*. *fumigatus* to persist in both azole-containing and azole-free environments [5].

## Which azole resistance mechanisms are found in *A*. *fumigatus*?

Several resistance mechanisms have been described in *A*. *fumigatus* involving mutations related to the azole target lanosterol 14α-demethylase (Cyp51A), HapE [8], Hmg1 [9], overexpression of efflux transporters, and increased target enzyme expression [10]. The diversity of resistance mechanisms underscores the potential of *A*. *fumigatus* to adapt, and it is likely that more currently uncharacterized mechanisms exist, as in 20%–50% of resistant phenotypes, a resistance mutation cannot be identified [10]. Cyp51A target enzyme alterations are the most commonly encountered resistance mechanisms in *A*. *fumigatus* isolates. The target enzyme changes include nonsynonymous single-nucleotide mutations, inducing amino acid substitutions at hotspots, such as M220, G54, and G138, or nonsynonymous resistance mutations in combination with tandem repeat (TR) in the gene promoter region, such as TR_34_/L98H and TR_46_/Y121F/T289A [10]. TRs have been shown to increase the expression of Cyp51A [11], and over the past years, novel TR variants have been reported, including variations in the number of repeat duplications (e.g., triplication of TR_34_ and TR_46_ [12, 6]), as well as the length of the repeat (e.g., TR_120_ [13]). As distinctly different Cyp51A alterations were found in different (sampling) environments, the concept of a patient and an environmental route of resistance selection was postulated [14].

## Which evidence supports a patient and environmental route of resistance selection?

Evidence for in-host resistance selection was provided through observations of patients presenting with aspergillus disease due to wild-type *A*. *fumigatus* that evolved to resistant phenotypes during triazole therapy. Microsatellite genotyping found wild-type and resistant isolates to be isogenic, supporting the selection of resistant clones in vivo [15]. A frequent characteristic of these patients is the presence of a pulmonary cavity. As *A*. *fumigatus* may undergo asexual sporulation in pulmonary cavities, it is likely that genetic variation increases through the generation of millions of spores. Resistant clones are then selected through triazole exposure. Nonsynonymous Cyp51A mutations are typically found in this setting, and a single aspergilloma may contain multiple resistance mutations [16]. Genotyping indicated that the diversity between resistant isolates with identical resistance mechanisms but recovered from different patients was notably higher than among resistant isolates found in the environment, supporting de novo in-host resistance selection rather than patient-to-patient transmission.

As triazole-resistant *A*. *fumigatus* was also encountered in patients without previous triazole exposure, the possibility of an environmental route was postulated [14,17]. Most of these patients were diagnosed with triazole-resistant IA, and the resistance mechanisms predominantly involved TR-associated mechanisms [18,19]. Furthermore, as *A*. *fumigatus* exclusively forms hyphae in IA, the fungus does not benefit from genetic variation induced by spore production; thus, in-host resistance development is unlikely. Alternatively, patients may inhale inherently triazole-resistant *A*. *fumigatus* spores, supported by the fact that in every case with a wild-type *A*. *fumigatus* coinfection, the genotypes remain disparate [18]. Moreover, recovery of *A*. *fumigatus* isolates from the environment harboring the same TR-associated resistance mutations as patient isolates supported an environment route of resistance selection. Indeed, genotyping of clinical and environmental isolates harboring TR_34_/L98H or TR_46_/Y121F/T289A showed phylogenetic clustering, indicating a closer genetic relatedness between them than with wild-type control isolates. Furthermore, in a controlled experimental evolution design, many environmental azole fungicides were found to be active against *A*. *fumigatus* to very effectively select for resistance and, crucially, to cause cross-resistance to the medical triazoles in these populations without ever being exposed to them [20]. The characteristics of both routes of resistance selection are summarized in Table 1.

**Table 1:** Characteristics of the patient and environmental route of resistance selection in *A*. *fumigatus*.

Patient route	Environmental route
Resistance is primarily found in patients with a pulmonary cavity	Resistance is found in all aspergillus diseases: invasive aspergillosis, ABPA, chronic colonization, aspergilloma
Recent or ongoing triazole therapy	Two-thirds of patients are triazole naïve
Isolates may have a fitness cost in culture	Isolates have no apparent fitness cost
Isogenic wild-type and resistant isolates may be found in consecutive samples from the same patient	Isogenic wild-type and resistant isolates are not found in consecutive samples from the same patient
*Resistance mutations include single mutations in the *Cyp51A* gene and non-Cyp51A (unknown) mutations	*Resistance mechanisms include single mutations in the *Cyp51A* gene combined with tandem repeats in the gene promoter
*Resistance mutations are found in clinical samples only	*Resistance mutations are found both in clinical samples and in the environment
Clinical samples may contain multiple different resistance mutations	Clinical samples generally contain one single-resistance mechanism but may also harbor wild-type isolates
Genetic typing shows high diversity in resistant isolates from different patients	Genetic typing shows low diversity in resistant isolates from different patients

*These statements are challenged by recent studies.

Abbreviation: ABPA, allergic bronchopulmonary aspergillosis.

## Which observations challenge the distinct association between specific resistance mutations and the route of resistance selection?

Although numerous studies have reported the presence of TR_34_/L98H, TR_53_, and TR_46_/Y121F/T289A in clinical and environmental samples, single-resistance mutations have also been found in settings not consistent with in-host resistance selection: the environment and from triazole-naïve patients. A wide variety of single-resistance mutations have been cultured from the environment, including single-resistance mutations that were also found in triazole-treated patients (Table 2). Furthermore, recently, a CPA patient was reported who developed triazole resistance in an *A*. *fumigatus* isolate harboring TR_120_ in the *Cyp51A* promoter [13]. The finding was supported by whole-genome sequencing data, illustrating that the resistant and a previously cultured wild-type isolate were isogenic, and other potential resistance mutations in the resistant isolate were absent. Thus, the selection of TR-associated resistance mechanisms may not be exclusive to the environment [13]. This evidentially challenges the exclusiveness of the association between resistance mechanism and route of resistance selection.

**Table 2:** Single-resistance mutations in triazole-resistant *A*. *fumigatus* recovered from azole-naïve patients and environmental samples.

Origin and year*	Geographic region	Sample	Azole exposure	*Cyp51A* resistance mechanisms^§^ (# of isolates)	Resistance phenotype	Comment	Reference
**Clinical**							
2006–2009	France	Clinical	None	G432S (1)	ITC R, PCZ S, VCZ S		[21]
2010	Germany	Pulmonary sample	None	M220L (1)	ITC R, PCZ R, VCZ S		[22]
2011–2015	France	Clinical	No systemic antifungal treatment in last 6 months	A284T (1); H285Y (1)	ITC S, PCZ R, VCZ S ITC R, PCZ R, VCZ R	Also isolates reported with changes at F46Y; M172V; N248T; D255E; E427K; in different combinations giving slightly elevated PCZ MICs	[23]
2013	Argentina	Cornea surface	None	G54E (1)	ITC R, PCZ S, VCZ S		[24]
2014	France	Pulmonary sample	None	F219I (1)	ITC R, PCZ R, VCZ S		[25]
2014*	Unknown	Pulmonary sample	None	Y121F (1)	ITC s, PCZS, VCZ R,		[26]
2013–2014	India	Pulmonary sample	None	P216L (3) G54R (1) Y431C (2)	ITC R, PCZ S, VCZ S ITC R, PCZ S, VCZ R ITC R, PCZ S/R, VCZ S		[27]
**Environmental**							
2005*	Switzerland	Soil	n.a.	Unknown (1), F46Y, M172V, E427K (1) ^§^	ITC S, VCZ R ITC R, VCZ S	In addition, several environmental isolates with ITC MIC 2–8 mg/l were reported without sequence information	[28]
2007	the Netherlands	Soil	n.a.	Unknown (3) F46Y/M172V/E427K^§^ (2)	ITC R, PCZ S, VCZ R; ITC R, PCZ S, VCZ S; ITC S, PCZ S, VCZ R; ITC S, PCZ S, VCZ R		[29]
2009–2011	Great Britain	Soil, Air	n.a.	Unknown (2)	ITC R, PCZ R, VCZ S-I		[30]
2011–2015	France	Dust, Patients home	n.a.	H285Y (1)	ITC R, PCZ R, VCZ R	Also, isolates reported with SNPs at F46Y; M172V; N248T; D255E; E427K; in different combinations, giving slightly elevated PCZ MICs	[23]
2011–2012	Italy	Soil	n.a.	Unknown (1), F46Y/M172V/ N248T/D255E (1) ^§^	ITC R, PCZ R, VCZ S; ITC R, PCZ R, VCZ I		[31]
2012–2013	Germany	Soil	n.a.	G54A (2) M220I (1)	ITC R, PCZ R, VCZ S; ITC R, PCZ R, VCZ R		[32]
2013–2014	Tanzania, Roemania, India	Soil	n.a.	G54E (21)	ITC R, PCZ R, VCZ S		[33]
2014–2016	Taiwan	Soil, Air	n.a.	WT cyp51A or SNPs (5)	ITC S-R, PCZ R, VCZ S-R		[34]
2014–2017	Italy	Soil	n.a.	G54E	ITC R, PCZ R, VCZ S		[35]
2015	Switzerland	Soil	n.a.	G54R	ITC R, PCZ S, VCZ S		[36]
2015–2016	France	Soil	Propiconazole and tebuconazole	Unknown (1), P216L (1)	ITC R, PCZ R, VCZ R; ITC R, PCZ R, VCZ S		[37]
2017*	Colombia	Soil	n.a.	Unknown	ITC R, VCZ S		[38]

*Year of publication, as date of sampling was not provided.

^§^Amino acid alterations that are reported in azole-susceptible and -resistant isolates.

**Abbreviations:** I, intermediate; ITC, itraconazole; PCZ, posaconazole; R, resistant; S, susceptible; VCZ, voriconazole.

## How can the emergence of single-resistance mutations in the environment and TR-mutations in patients be explained?

Although the conditions present in the human host may facilitate the selection of single-resistance mutations and exposure of *A*. *fumigatus* to azole fungicides the development of mechanisms with TRs, the distinction between a patient route and environmental route is artificial. Rather than the location where resistance is selected, characteristics of the fungus and its environment will determine the supply of mutations and subsequent selection. Factors that are critical include the fungal effective population size, mode of reproduction, biochemical characteristics of the antifungal drug, and concentration, which ultimately determine whether resistant clones will emerge. Although certain mechanisms may dominate in specific habitats, over time we may not be able to reliably derive the route of resistance selection based only on the resistance mechanism found due to increasing diversity of resistance mechanisms.

## What is the relevance of the origin of resistance mutations?

The origin of azole resistance selection is important for management of patients with *Aspergillus* diseases. Although the development of azole resistance can be anticipated in patients on chronic azole therapy [39], environmental resistance can affect any patient who is prone to develop *Aspergillus* disease irrespective of their azole treatment history [14,19]. In patients with azole-resistant IA, early detection of resistance was found to be critical for survival [4]. As most patients with IA are culture negative, molecular tests have been developed and validated that allow detection of resistance mutations directly in clinical samples [40]. Thus far, commercial PCR tests include only TR-associated resistance targets [40], and as a consequence, other azole resistance mechanisms will not be detected. Therefore, information on the range and frequency of resistance mutations that can be encountered in the environment is important for development of reliable molecular tests. Furthermore, in order to halt the global spread of environmental resistance mutations, strategies are needed that prevent or reduce the burden of resistance induced by fungicide use.

## What are our next steps?

The potentially fading boundaries between patient and environmental routes of resistance selection is an incentive to focus on the conditions that facilitate the development, selection, and spread of resistance genotypes, which is best approached through multidisciplinary collaboration and from a one-health perspective. In a recent study, targeted environmental sampling showed high levels of azole-resistant *A*. *fumigatus* in stockpiles of flower bulb waste, green waste, and wood chippings [41]. The common feature of these habitats was the ability of *A*. *fumigatus* to thrive and reproduce, and the presence of azole fungicide residues [41]. Such insights are critical to identify practices that confer a high risk for resistance selection and to design interventions that reduce the burden of environmental resistance. Furthermore, the common features that characterize the “hotspot” for resistance selection could be helpful to identify additional hotspots. On a global scale, understanding the dynamics and spread of resistance is essential to determine whether azole resistance mutations have arisen through a single event and subsequent spread or continue to emerge in different geographic regions.

Little is known about the origin of single-resistance mutations that are recovered from the environment. It could be that specific environmental conditions exist that facilitate the emergence of these resistance mutations. Alternatively, humans harboring resistant *A*. *fumigatus* might contaminate their environment. Indeed, a recent study indicated possible aerosol patient-to-environment transmission of *A*. *fumigatus* in chronically colonized patients [42]. An evolutionary trajectory of the fungus that involves a phase in the human lung could change the way we manage patients with chronic lung colonization.

Clearly, azoles play a major role in the world’s food production, and at least until alternatives to chemical use are fully developed, our goal should be to retain the potential of using this class for agricultural applications. But this should not go at the expense of the availability of azoles for patient treatment. Thus, a better understanding of resistance selection dynamics and spread and risk factors associated with resistance selection will help us to design measures that prevent or control the problem in the environment as well as in patients and will improve our ability to diagnose and manage resistance in human infection.
